# Differences in treatment and outcomes between young and older patients with colorectal cancer liver metastases: an AmCORE-based study

**DOI:** 10.1016/j.esmogo.2026.100435

**Published:** 2026-11-09

**Authors:** J.F.E. Vedder, H.H. Schulz, R.-J. Swijnenburg, M.R. Meijerink, K.S. Versteeg, T.E. Buffart

**Affiliations:** 1 Department of Medical Oncology, Amsterdam UMC, Amsterdam, The Netherlands; 2 Cancer Center Amsterdam, Amsterdam UMC, Amsterdam, The Netherlands; 3 Department of Radiology and Nuclear Medicine, Amsterdam UMC, Amsterdam, The Netherlands; 4 Department of Surgery, Amsterdam UMC, Amsterdam, The Netherlands

## Abstract

**Background:**

The incidence of early-onset colorectal cancer (eoCRC, <50 years) is rising, whereas late-onset CRC (loCRC, ≥70 years) is increasingly prevalent due to demographic shifts. CRC commonly metastasizes to the liver, impacting progression-free survival (PFS) and overall survival (OS). Current guidelines are mainly based on patients aged 50-70 years, and little is known about treatment and outcomes in younger and older patients with colorectal liver metastases (CRLM).Therefore, we compared treatment strategies and outcomes between eoCRC and loCRC patients with CRLM.

**Patients and methods:**

Patients receiving their first local treatment of CRLM between 2018 and 2023 at Amsterdam UMC were extracted from the AmCORE registry and subdivided into three age groups: <50, 50-69, and ≥70 years. Primary and secondary endpoints were PFS and OS, respectively. Firth logistic regression evaluated factors influencing treatment choice.

**Results:**

In total, 351 patients were included: 31 aged <50, 164 aged 50-69, and 156 aged ≥70. Younger patients received significantly more induction chemotherapy before first local treatment than those aged ≥70 and 50-69 (*P* < 0.001) and presented more frequently with a higher number of CRLM (*P* = 0.026). PFS was significantly shorter in patients <50 (*P* = 0.019), whereas OS did not differ significantly between age groups (*P* = 0.22). A larger number and size of CRLM were associated with an increased likelihood of induction systemic treatment.

**Conclusions:**

Despite receiving more intensive treatment, younger patients did not show better outcomes: PFS was poorer and OS was similar compared with older patients. This may be explained by greater disease burden at presentation rather than intrinsically aggressive tumor biology.

## Highlights

• Young patients with CRLM received more induction chemotherapy.
• Young patients had shorter PFS despite intensified treatment.
• Older patients had similar OS despite less aggressive therapy.
• Number and size of liver metastases were associated with induction treatment.

## Graphical abstract

**Figure:**
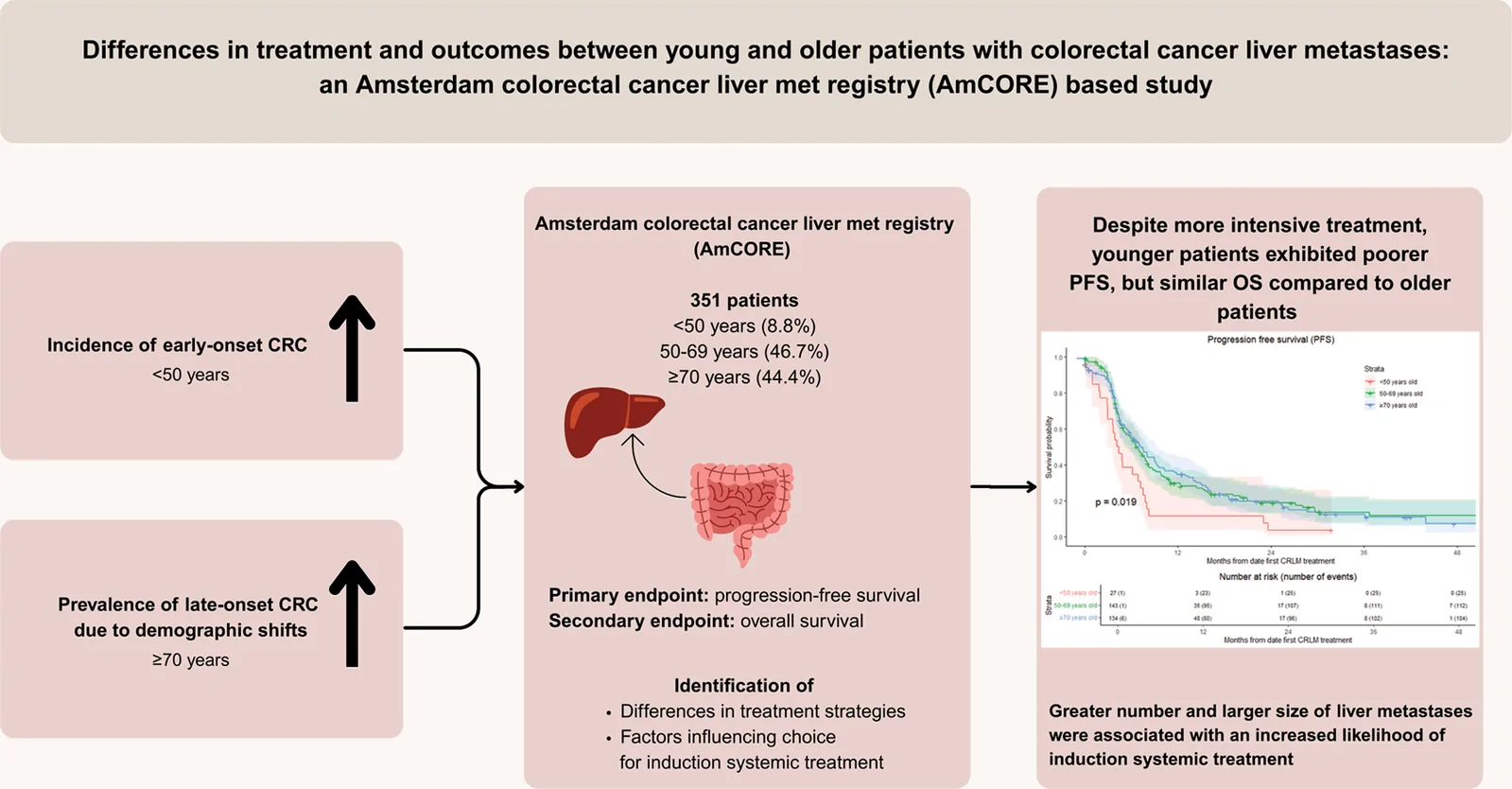

## Introduction

Colorectal cancer (CRC) accounts for 9.6% of the global annual cancer incidence. In 2024, CRC was one of the leading causes of cancer-related mortality, with a death rate of 4.7%, mostly due to metastatic disease.^1^, ^2^ CRC is typically a disease of adults over 50 years of age, with the majority of cases occurring in individuals aged ≥70 years (late-onset CRC, loCRC).^3^ Recent data, however, indicate a rising incidence of sporadic, nonhereditary CRC in individuals under 50 years of age [early-onset colorectal cancer (eoCRC)], particularly in high-income countries.^4^, ^5^ Importantly, CRC is the leading cause of cancer-related mortality in young men and the second leading cause among young women.^6^ Together, this has led to a clinically relevant distinction between eoCRC, average-onset CRC (50-69 years, aoCRC), and loCRC, each with potentially distinct disease trajectories and treatment needs. Throughout the course of the disease, 50%-60% of patients develop distant metastases, most frequently in the liver (80%-90%).^7^ Colorectal liver metastases (CRLM) are often detected within the first 12 months after diagnosis of CRC but nearly 10% are diagnosed later in the course of the disease.^8^ When metastases are limited to the liver, local treatment, mostly by resection and/or ablation, is a potentially curative treatment with a 5-year survival rate of 51.2%-58%.^9^, ^10^

Although local treatment remains central in managing CRLM, for several well-established reasons, systemic therapy plays a vital role in the multidisciplinary approach. Firstly, when CRLM are not considered locally treatable, induction systemic therapy is indicated for downstaging, resulting in a locally treatable situation in 45%-58% of patients.^11^ Furthermore, arguments for administering systemic therapy in patients with upfront locally treatable disease include reducing procedural risk, especially in cases requiring major hepatectomy, or addressing poor prognostic factors such as more than five CRLM, synchronous metastases, rat sarcoma viral oncogene homolog (RAS) or v-Raf murine sarcoma viral oncogene homolog B1 (BRAF) mutations, or a right-sided primary tumor.^12^ Lastly, in patients with poor prognostic factors, induction systemic therapy may help to identify those patients with rapidly progressive, treatment-resistant disease who may be spared unnecessary and potentially harmful invasive procedures.^13^

Importantly, treatment decisions for CRLM are not solely based on tumor characteristics but also on patient-related factors, with age possibly playing a substantial role. Treatment guidelines are based on outcomes of clinical trials mainly including patients aged 50-70 years,^12^ but eoCRC and loCRC patients are generally underrepresented. Consequently, evidence supporting the use of the same treatment regimens in eoCRC or loCRC patients with CRLM is lacking. Despite guideline-based treatment approaches across age groups, recent reports suggest that younger patients are treated more aggressively. They are two to four times more likely to receive adjuvant systemic treatment of the primary tumor than older patients, regardless of the disease stage at diagnosis.^14^, ^15^, ^16^ In contrast, older patients are at risk of undertreatment. When indicated, a smaller percentage of older patients with CRLM receive perioperative chemotherapy than younger patients, despite its association with improved oncological outcomes.^17^ The complexity of treating patients with CRLM, coupled with the evolving patient population, necessitates a more thorough understanding of the specific differences in applied treatment regimens and outcomes of eoCRC and loCRC patients.

This study aimed to assess the differences in current treatment regimens and clinical outcomes for eoCRC and loCRC patients who have been treated for CRLM.

## Materials and methods

### Patient selection and data collection

This study was conducted at the Amsterdam University Medical Center in The Netherlands, a tertiary referral center specialized in hepatobiliary and gastrointestinal malignancies. Data were obtained from the AmCORE (Amsterdam colorectal cancer liver met registry) database, which includes prospectively collected information on patients diagnosed with CRLM who received at least one local treatment for CRLM. All patients provided informed consent during a clinical visit. The study was approved by the affiliated institutional review board (METc 2021.01.0121).

Patients were included in this study if they had received the first local treatment for CRLM between 1 January 2018 and 31 December 2023. Follow-up was completed until 1 February 2025. Patients with missing data regarding disease characteristics, treatment characteristics, and follow-up were excluded (Figure 1).

**Figure 1:**
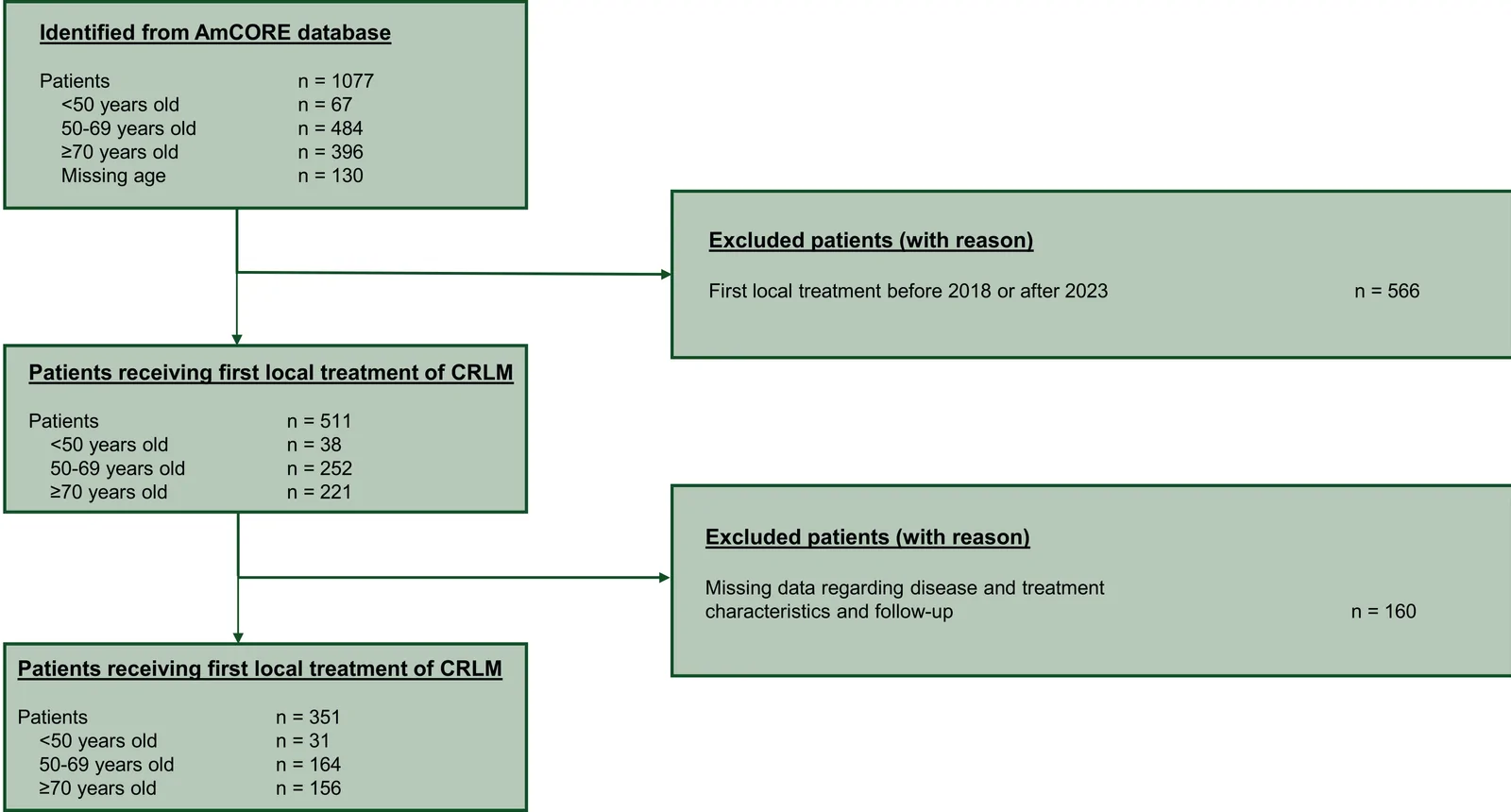
**Flowchart of included and excluded patients**.

Clinical data were collected retrospectively through a review of electronic patient records and comprised patient-, disease-, and treatment-related characteristics. Stage at diagnosis was based on pathological (pT/pN) stage in patients who underwent resection of the primary tumor and on clinical (cT/cN) stage otherwise. CRLM diagnosed within 6 weeks of the primary tumor were defined as synchronous CRLM, those diagnosed between 6 weeks and 1 year as early metachronous, and those diagnosed ≥1 year after the primary tumor diagnosis as late metachronous.^18^ Best-observed response to systemic treatment was classified as complete response, partial response (PR), stable disease, or progressive disease.

### Statistical analysis

Baseline characteristics were compared between three groups: patients <50 years of age (eoCRC), patients between 50 and 69 years of age (aoCRC), and patients ≥70 years of age (loCRC). Categorical variables were reported as absolute numbers as well as frequencies (in percentage). Continuous variables were reported as median with interquartile range (IQR). Differences in baseline characteristics across age groups were assessed using Pearson’s chi-square test or Fisher’s exact test for categorical variables, and Mann–Whitney *U* test or Kruskal–Wallis test for continuous variables. *Post hoc* pairwise comparisons were carried out to examine differences between age groups, with the Holm–Bonferroni correction applied to account for multiple testing.

Kaplan–Meier survival curves with the log-rank test were used to estimate and compare progression-free survival (PFS) and overall survival (OS). The primary endpoint in the PFS curves was defined as time to disease recurrence (recurrence of locally treated CRLM, the appearance of new lesions in the liver or other distant metastases) from the date of the local treatment of CRLM, rather than from diagnosis of CRLM, in order to allow evaluation of local treatment. The primary endpoint in the OS curves was defined as time to death from the date of diagnosis of first CRLM. Data were right-censored for those still alive during the last follow-up. *P* values < 0.05 were considered statistically significant.

Possible confounders were specified a priori based on clinical plausibility and literature (sex, comorbidities, primary tumor location, RAS/BRAF mutation status, mismatch repair [MMR] status, T stage, N stage, synchronous or early- or late-metachronous CRLM, number of CRLM at diagnosis, and diameter of largest CRLM at diagnosis). Starting from a Cox model with age category as the exposure (reference 50-69 years), each possible confounder was added one at a time. A variable was retained as a confounder when addition of the variable changed the age hazard ratio(s) by ≥10% relative to the crude model. Because age categories consist of three groups, the maximum relative change across contrasts was used. Hazard ratios (HRs) and 95% confidence intervals (95% CIs) were reported. Sensitivity analysis was carried out using a propensity-score–weighted Cox model, weighting for the likelihood of receiving systemic treatment before local treatment based on sex, comorbidities, primary tumor location, BRAF/RAS/MMR status, T and N stage, timing of detection of CRLM, number of CRLM at diagnosis, and diameter of largest CRLM. Firth logistic regression was used to evaluate the impact of variables on the choice of induction systemic treatment. Odds ratios (OR) and 95% CIs were reported. All statistical analyses were conducted using R Version 4.3.3 (R Foundation, Vienna, Austria).^19^

## Results

### Patient, disease-, and treatment-related characteristics

Baseline patient-, disease-, and treatment-related characteristics are outlined in Table 1. In total, 351 patients were included, of whom 31 (8.8%) were eoCRC, 164 (46.7%) were aoCRC, and 156 (44.4%) were loCRC. The median age was 66 years (IQR 57-75). The prevalence of major comorbidities was significantly higher in loCRC (16.1%) compared with aoCRC (5.9%) and eoCRC patients (0%) (*P* < 0.001). In loCRC, the primary tumor was significantly more frequently left-sided (32.9%) compared with aoCRC and eoCRC (*P* = 0.030). eoCRC patients presented significantly more often with synchronous CRLM (71%) compared with aoCRC (55.5%) and loCRC (53.2%) (*P* = 0.027). eoCRC patients had a significantly higher number of CRLM at diagnosis (4, IQR 1.5-9) compared with aoCRC (2, IQR 1-5) and loCRC (2, IQR 1-4) (*P* = 0.030). No significant differences between the age groups were observed in the diameter of the largest CRLM or in the molecular profile.

**Table 1:** Baseline characteristics

Characteristics		Total *N* = 351 (100%)	<50 years *n* = 31 (8.8%)	50-69 years *n* = 164 (46.7%)	≥70 years *n* = 156 (44.4%)	*P* value
Patient-related characteristics						
Sex	Male	234 (66.7)	18 (58.1)	101 (61.6)	115 (73.7)	
	Female	117 (33.3)	13 (41.9)	63 (38.4)	41 (26.3)	0.040^a^
Age (years)		67 (57-75)	43 (41-47)	60 (54-64)	76 (72-80)	
ASA physical status	1	12 (3.6)	1 (3.3)	7 (4.5)	4 (2.8)	
	2	226 (68.5)	25 (83.3)	114 (73.5)	87 (60)	
	3	91 (27.6)	4 (13.3)	34 (21.9)	53 (36.6)	
	4	1 (0.3)	0	0	1 (0.7)	0.018^b^
Comorbidities	None	142 (47)	21 (72.4)	83 (61)	38 (27.7)	
	Minimal	130 (43)	8 (27.6)	45 (33.1)	77 (56.2)	
	Major	30 (9.9)	0	8 (5.9)	22 (16.1)	<0.001^b^
BMI (kg/m^2^)		25 (22.5-27.7)	25.6 (23.4-27.6)	25.0 (22-27.6)	24.8 (22.9-27.8)	0.779^c^
Disease-related characteristics						
Primary tumor location	Rectum	115 (32.9)	12 (38.7)	53 (32.3)	50 (32.3)	
	Left-sided colon	89 (27.1)	6 (19.4)	31 (18.9)	51 (32.9)	
	Right-sided colon	147 (42)	13 (41.9)	80 (48.8)	54 (34.8)	0.030^a^
Molecular profile^d^	RAS					
	Wild-type	100 (30.4)	12 (41.4)	49 (31.4)	39 (27.1)	0.536^b^
	Mutation	91 (27.7)	10 (34.5)	52 (33.3)	29 (20.1)	
	Unknown	138 (41.9)	7 (24.1)	55 (35.3)	76 (52.8)	
	BRAF					
	Wild-type	178 (54.3)	21 (72.4)	94 (60.6)	63 (43.8)	0.912^b^
	Mutation	12 (3.7)	1 (3.4)	6 (3.9)	5 (3.5)	
	Unknown	138 (42.1)	7 (24.1)	55 (35.5)	76 (52.8)	
	MMR					
	MMR proficient	262 (77.1)	30 (96.8)	140 (87)	92 (62.2)	0.527^b^
	MMR deficient	7 (2.1)	0	3 (1.9)	4 (2.7)	
	Unknown	71 (20.9)	1 (3.2)	18 (11.2)	52 (35.1)	
Extrahepatic metastases at diagnosis	Yes	33 (9.5)	2 (6.5)	19 (11.7)	11 (7.1)	
	No	315 (90.5)	29 (93.5)	144 (88.3)	143 (92.9)	0.394^b^
CEA at diagnosis (μg/l)		8 (3-23)	5 (2-20)	9 (4-32)	8 (3.8-19.2)	0.286^c^
FONG-score	1	55 (50.5)	4 (12.9)	31 (60.8)	20 (40)	
	2	39 (35.8)	4 (12.9)	13 (25.5)	22 (44)	
	3	14 (12.8)	0	7 (13.7)	7 (14)	
	4	1 (0.9)	0	0	1 (2)	
	5	0	0	0	0	0.226^b^
cT/pT stage	T1	10 (3.1)	0	3 (2.0)	7 (4.9)	0.547^b^
	T2	28 (8.8)	2 (6.7)	15 (10.1)	11 (7.7)	
	T3	214 (66.9)	20 (66.7)	95 (64.2)	99 (69.7)	
	T4	68 (21.2)	8 (26.7)	35 (23.6)	25 (17.6)	
cN/pN stage	N0	106 (33.2)	4 (12.9)	48 (32.9)	54 (38)	0.032^a^
	N1	147 (46.1)	16 (51.6)	65 (44.5)	66 (46.5)	
M stage	N2	66 (20.7)	11 (35.5)	33 (22.6)	22 (15.5)	0.132^a^
	M0	150 (44)	8 (27.6)	69 (43.4)	73 (47.7)	
	M1	191 (46)	21 (72.4)	90 (56.6)	80 (52.3)	
(Neo)adjuvant treatment of primary tumor	Neoadjuvant chemotherapy	21 (6)	4 (12.9)	12 (7.3)	5 (3.2)	
	Adjuvant chemotherapy	42 (11.1)	1 (2.4)	25 (15.2)	18 (11.6)	
	Monotherapy	9 (22.4)	0	3 (12)	6 (37.5)	
	Doublet therapy	31 (77.6)	1 (100)	22 (88)	8 (50)	
	Chemoradiotherapy	40 (11.4)	8 (25.8)	22 (13.4)	10 (6.5)	
	Radiotherapy	30 (8.6)	3 (9.7)	9 (5.5)	18 (11.6)	
	None	220 (62.9)	16 (49.2)	100 (61)	104 (67.1)	<0.001^a^
Surgical resection of primary tumor	Yes	313 (89)	26 (84)	148 (90)	139 (89)	0.500^b^
	No	38 (11)	5 (16)	16 (10)	17 (11)	
Timing of surgical resection of primary tumor	Before local treatment CRLM	237 (76)	13 (50)	110 (74)	114 (82)	0.003^b^
	After local treatment CRLM	64 (20)	13 (50)	32 (22)	19 (14)	
	Concurrent	12 (4)	0	6 (4)	6 (4)	
Time between resection of primary tumor and CRLM (months)	Before local treatment CRLM	10 (5-21)	7 (5-8)	11 (5-21)	9 (4-22)	0.300^c^
	After local treatment CRLM	2 (1.5-4.2)	2.1 (1.6-3.0)	1.9 (1.5-3.9)	2.7 (1.6-3.0)	0.700^c^
Timing of CRLM diagnosis	Synchronous	196 (55.8)	22 (71)	91 (55.5)	83 (53.2)	
	Early metachronous	66 (18.8)	8 (25.8)	33 (20.1)	25 (16)	
	Late metachronous	89 (25.4)	1 (3.2)	40 (24.4)	48 (30.8)	0.027^a^
Number of CRLM at diagnosis		2 (1-5)	4 (1.5-9)	2 (1-5)	2 (1-4)	0.030^c^
Diameter of the largest CRLM (mm)		25 (18-37)	26 (20-33)	26 (18-40)	25 (18-35)	0.727^c^
CRLM treatment-related characteristics						
Type of local treatment	Resection	115 (35.9)	8 (29.6)	62 (40.3)	45 (32.4)	
	Thermal ablation	127 (39.7)	11 (40.7)	51 (33.1)	65 (46.8)	
	Resection and thermal ablation	78 (24.4)	8 (29.6)	41 (26.6)	29 (20.9)	0.174^a^
Recurrent local treatment	Yes	150 (42.7)	18 (58.1)	70 (42.7)	62 (39.2)	
	No	201 (57.3)	13 (41.9)	94 (57.3)	96 (60.8)	0.074^a^
Total number of local treatment sessions	Median (minimum–maximum)	1 (1-8)	2 (1-8)	1 (1-8)	1 (1-5)	0.027^c^
Number CRLM treated in first session		2 (1-4)	3 (1-8.5)	2 (1-5)	2 (1-4)	0.550^c^
Systemic treatment-related characteristics						
Systemic treatment at any time in disease course	Yes	217 (61.8)	27 (87.1)	119 (72.6)	71 (45.5)	
	No	134 (38.2)	4 (12.9)	45 (27.4)	85 (54.5)	<0.001^a^
Induction systemic treatment before first local treatment of CRLM	Yes	119 (34)	18 (58.1)	66 (40.5)	34 (21.8)	
	No	231 (66)	12 (38.7)	97 (59.5)	122 (78.2)	<0.001^a^

Categorical variables are reported as absolute numbers of patients (%); continuous variables are reported as median (IQR).

ASA, American Society of Anesthesiologists Score; BMI, body mass index; BRAF, v-Raf murine sarcoma viral oncogene homolog B1; CEA, carcinoembryonic antigen; cN stage, clinical N stage; CRLM, colorectal liver metastases; cT stage, clinical T stage; Fong-score, prognostic tool used to predict recurrence after hepatic resection for colorectal liver metastases; IQR, interquartile range; MMR, mismatch repair; pN stage, pathological N stage; pT stage, pathological T stage; RAS, rat sarcoma viral oncogene homolog.

a Pearson’s chi-square test.

b Fisher’s exact test.

c Kruskal–Wallis test.

d Reported *P* values reflect the comparison between wild-type and mutated groups only; patients with unknown RAS, BRAF, or MMR status were not included in this analysis.

### Treatment-related characteristics of the primary tumor and local treatment of CRLM

eoCRC patients received significantly more (neo)adjuvant treatment of the primary tumor (50.8%) compared with aoCRC (39%) and loCRC (32.9%) (*P* < 0.001). This accounts for neoadjuvant chemotherapy in colon cancer as well as for chemoradiotherapy in rectal cancer (*P* = 0.003 and *P* = 0.027, respectively). loCRC patients more often received radiotherapy only for rectal cancer compared with eoCRC and aoCRC (*P* = 0.027) (Supplementary Tables S1 and S2, available at https://doi.org/10.1016/j.esmogo.2026.100435). Of all patients, 313 (89%) underwent resection of the primary tumor, which was similar in all age groups (*P* = 0.500) (Table 1). Resection of the primary tumor preceding local treatment of CRLM became more common with increasing age (82% in loCRC versus 74% in aoCRC and 50% in eoCRC) (*P* = 0.003). There were no significant differences in type of local treatment of CRLM (e.g. thermal ablation, resection, or combined procedures) between the age groups (*P* = 0.174). Recurrent local treatments of CRLM were carried out in 58.1% of eoCRC patients compared with 42.7% and 39.2% in aoCRC and loCRC, respectively (*P* = 0.074). Sex and the number of CRLM treated in the first session showed a significant overall difference between age groups, but, unlike the other variables, no significant pairwise differences were identified in *post hoc* analyses (Supplementary Table S3, available at https://doi.org/10.1016/j.esmogo.2026.100435).

### (Induction) systemic treatment-related characteristics

Throughout the disease course, significantly more eoCRC patients received systemic treatment (87.1%) compared with aoCRC (72.6%) and loCRC (45.5%) (*P* < 0.001). Induction systemic treatment before the first local treatment of CRLM was also more frequently administered in eoCRC patients (58.1%) compared with aoCRC and loCRC (40.5% and 21.8%, respectively) (*P* < 0.001). Triplet chemotherapy as induction systemic treatment, consisting of 5-fluorouracil, oxaliplatin, and irinotecan with or without the addition of bevacizumab, was less frequently administered in loCRC patients (8.8%) compared with aoCRC and eoCRC (25.8% and 21.1%, respectively) (*P* = 0.007). The majority of patients in all age groups had a PR to systemic treatment (85.7%) and the best-observed response was similar across the three different age groups (*P* = 0.374) (Figure 2 and Supplementary Table S4, available at https://doi.org/10.1016/j.esmogo.2026.100435).

**Figure 2:**
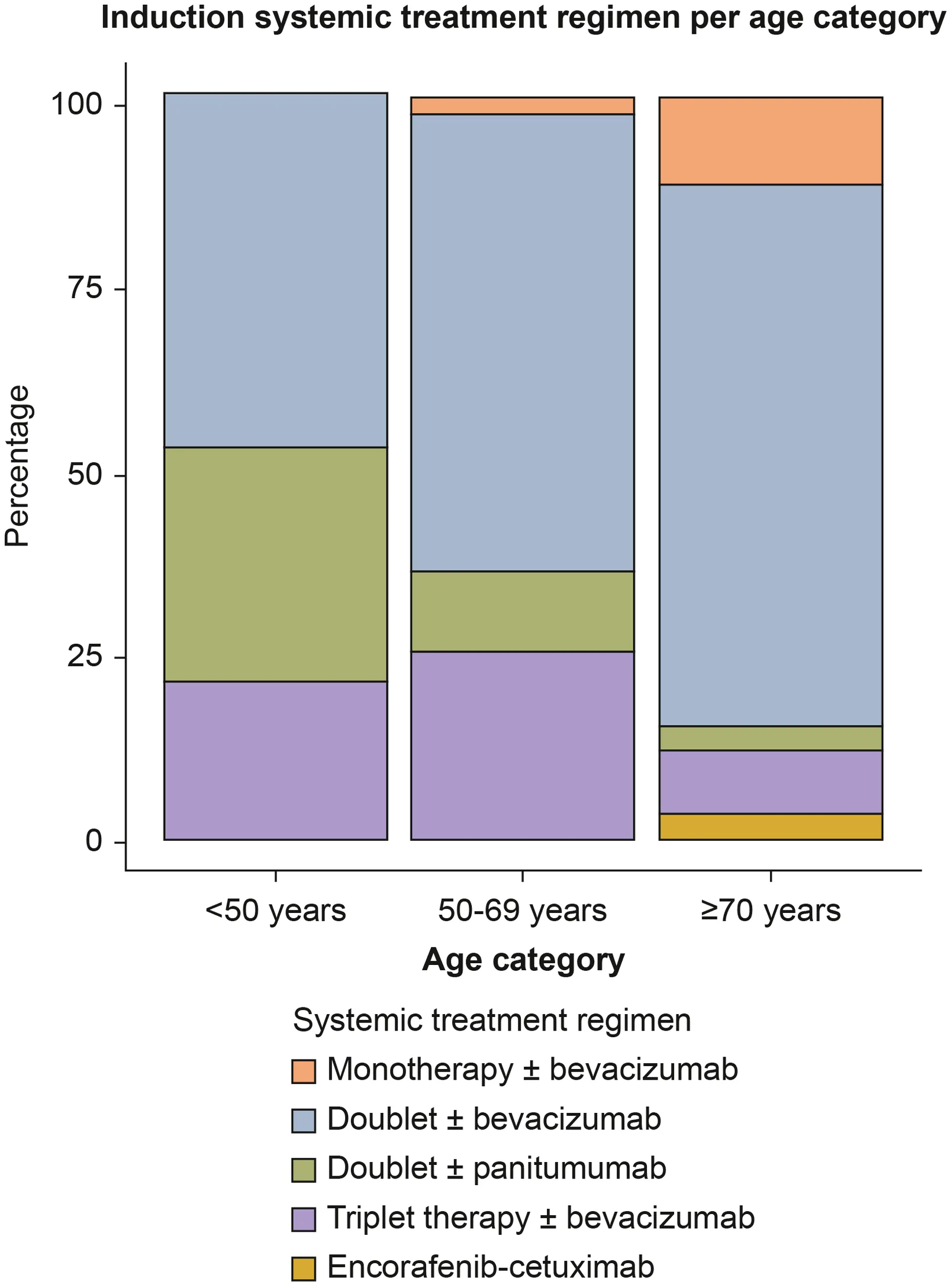
**Induction systemic treatment regimens.** Distribution of systemic treatment regimens by age category. The *x*-axis represents the three age categories. The *y*-axis shows the percentage of patients within each age group receiving a specific treatment. The colors in the bars indicate the type of systemic treatment received.

Subanalyses by age showed that across all age groups, patients receiving induction systemic treatment had a higher number of CRLM at diagnosis compared with those who did not (*P* < 0.001) (Supplementary Tables S5-S7, available at https://doi.org/10.1016/j.esmogo.2026.100435). This was evident for patients with synchronous as well as metachronous CRLM (Supplementary Table S8, available at https://doi.org/10.1016/j.esmogo.2026.100435). Furthermore, in aoCRC patients, the systemic therapy induction group more often presented with synchronous disease and had a larger CRLM size compared with the noninduction group (both *P* < 0.001). Among loCRC patients, induction systemic treatment was associated with right-sided colon tumors and larger CRLM size compared with those who did not receive induction treatment (*P* = 0.030 and *P* = 0.003, respectively).

### Subsequent treatment patterns after first local treatment of CRLM

Treatment after first local treatment of CRLM differed by age group (Supplementary Table S9, available at https://doi.org/10.1016/j.esmogo.2026.100435). Subsequent local treatment was more common in eoCRC than in aoCRC and loCRC patients (61% versus 45% versus 39%; *P* = 0.003). Palliative systemic therapy after first local treatment of CRLM was comparable across age groups. The number of systemic and subsequent local treatment sessions decreased with increasing age (*P* < 0.001 and *P* = 0.027, respectively). Among the 153 patients who underwent subsequent local treatment, both preceding systemic treatment (58% versus 44% versus 21%; *P* < 0.001) and transition to palliative systemic therapy thereafter (35% versus 30% versus 16%; *P* = 0.016) became less common with increasing age.

### PFS

The median PFS of the entire cohort was 6.6 months (95% CI 5.7-7.6), with a median follow-up of 31.1 months (95% CI 26.6-42.0). eoCRC patients had a significantly lower PFS compared with aoCRC and loCRC (*P* = 0.019) (Figure 3A and Supplementary Table S10, available at https://doi.org/10.1016/j.esmogo.2026.100435).

**Figure 3:**
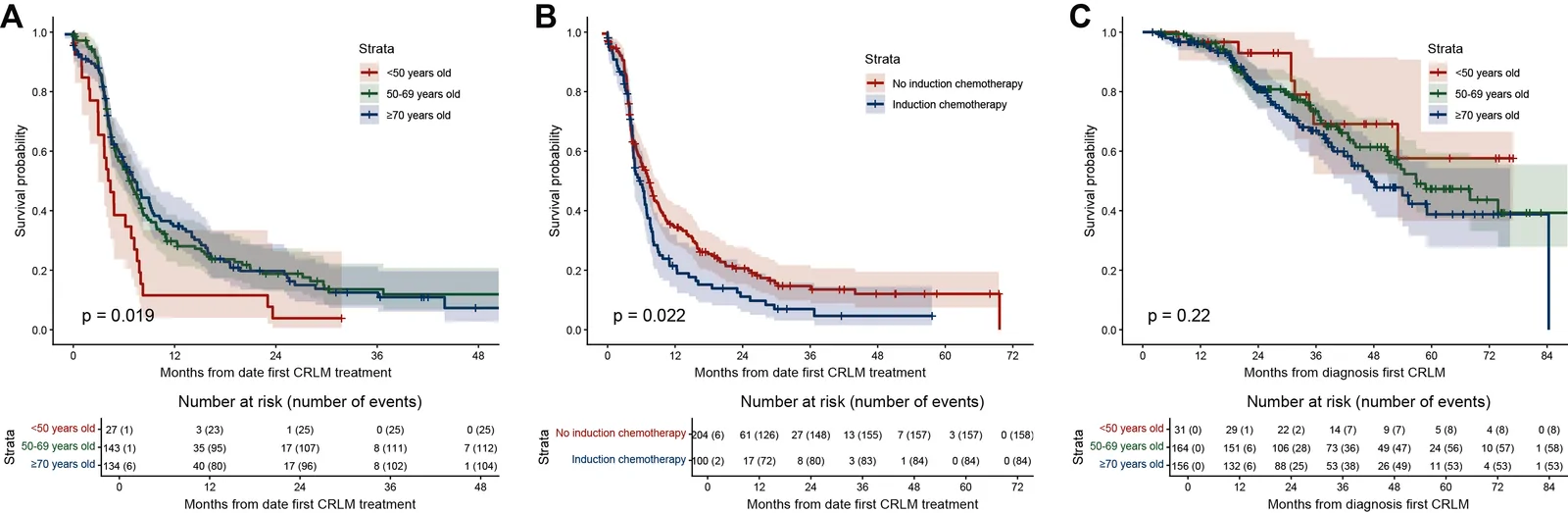
**Kaplan–Meier survival curves.** (A) PFS by age group (*P* = 0.019). Red indicates patients <50 years, green indicates patients 50-69 years, blue indicates patients ≥70 years. (B) PFS by induction chemotherapy (*P* = 0.022). Red indicates patients without induction chemotherapy; blue indicates patients with induction chemotherapy. (C) OS by age group (*P* = 0.22). Red indicates patients <50 years, green indicates patients 50-69 years, blue indicates patients ≥70 years. PFS, progression-free survival.

In patients who received induction chemotherapy, the median PFS was 5.6 months (95% CI 4.6-7.0) compared with 7.4 months (95% CI 6.0-9.1) in patients who did not (*P* = 0.022) (Figure 3B). When analyzing the three age categories separately, PFS only remained significantly different between these two groups in aoCRC patients (*P* = 0.015) (Supplementary Figure S1, available at https://doi.org/10.1016/j.esmogo.2026.100435).

### OS

At a median follow-up of 40.2 months (95% CI 37.8-46.4), the median OS of the entire cohort was 54.3 months (95% CI 50.8-NA), with a 1-year OS of 96.1% (95% CI 94.1-98.2), a 3-year OS of 70.2% (95% CI 64.8-76.0), and a 5-year OS of 44.6% (95% CI 37.3-53.3). There was no significant difference in OS between the three different age groups (*P* = 0.22) (Figure 3C).

Median OS of patients who received induction systemic treatment before the first local treatment was 51.2 months (95% CI 42.7-NA) compared with 59.0 months (95% CI 47.5—not reached) in patients who did not receive induction systemic treatment (*P* = 0.66) (Supplementary Figure S2, available at https://doi.org/10.1016/j.esmogo.2026.100435).

### Association of variables to OS

In univariable analysis, major comorbidities (HR = 2.09, 95% CI 1.17-3.73, *P* = 0.047), left-sided primary tumors (HR = 2.26, 95% CI 1.40-3.66, *P* = 0.003) and N2 disease (HR = 2.19, 95% CI 1.31-3.66, *P* = 0.012) were associated with OS. After adjustment for the detected confounders by multivariable analysis (comorbidities, T stage, and BRAF mutation status), N stage and timing of diagnosis of CRLM (synchronous or early- and late metachronous) were independently associated with OS (*P* = 0.015 and *P* = 0.036, respectively) (Supplementary Table S11, available at https://doi.org/10.1016/j.esmogo.2026.100435). In a propensity-score–weighted sensitivity analysis for the comparison between aoCRC and loCRC, neither age group (HR 0.86, 95% CI 0.45-1.64, *P* = 0.66) nor systemic treatment before local treatment (HR 0.90, 95% CI 0.47-1.73, *P* = 0.75) was significantly associated with OS, consistent with the primary multivariable analysis.

### Variables influencing the decision for induction systemic treatment

Compared with aoCRC patients, loCRC patients had 56% lower odds (OR = 0.44, 95% CI 0.18-1.05, *P* = 0.064) of receiving induction systemic treatment, whereas eoCRC patients had 78% higher odds of receiving induction systemic treatment (OR = 1.78, 95% CI 0.57-5.63, *P* = 0.321). Furthermore, a larger diameter of the largest CRLM and a higher number of CRLM at diagnosis were significantly associated with increased odds of receiving induction systemic treatment (OR = 1.02, 95% CI 1.00-1.05, *P* < 0.001; OR = 1.56, 95% CI 1.36-1.82, *P* < 0.001, respectively) (Supplementary Table S12, available at https://doi.org/10.1016/j.esmogo.2026.100435).

## Discussion

In this retrospective cohort study, we compared treatment and outcomes of patients with CRLM across three age groups. eoCRC patients received more intensive systemic therapy, including induction treatment, triplet regimens, and (neo)adjuvant treatment of the primary tumor, yet had similar OS and even shorter PFS than aoCRC and loCRC patients, suggesting that factors beyond treatment intensity may drive their poorer prognosis.

Our results do not necessarily substantiate an intrinsically more aggressive tumor biology in eoCRC patients but may point to later detection as a key driver of aggressive behavior. eoCRC patients more often presented with synchronous CRLM and a greater number of CRLM, possibly reflecting diagnostic delays and the absence of screening below the age of 55 in The Netherlands.^20^, ^21^, ^22^, ^23^, ^24^, ^25^ Both are known risk factors for recurrence^26^, ^27^, ^28^ and plausibly explain the shorter PFS despite more intensive treatment. The intensified treatment of younger patients, consistent with existing literature, is thus better understood as a response to greater disease burden at presentation than as evidence of aggressive biology *per se*.^14^, ^15^, ^16^, ^21^, ^22^, ^23^, ^29^ Nevertheless, a biological contribution cannot be excluded, as younger patients received more local treatments and systemic treatment sessions, which may either reflect greater physiological reserve or more aggressive disease necessitating high-intensity treatment.

Notably, right-sided primary tumors were most prevalent in our eoCRC patients, whereas eoCRC is generally predominated by left-sided colon or rectal cancer. This may reflect selection for liver-directed treatment, given the propensity of right-sided tumors to metastasize to the liver via portal venous drainage.^30^

loCRC patients more frequently presented with (late) metachronous CRLM and fewer CRLM, which may partially explain their comparable OS despite less intensive treatment. This is in line with studies showing similar survival with deintensified treatment in older patients.^31^, ^32^ The higher proportion of metachronous CRLM in older patients likely reflects regular postresection surveillance,^14^, ^17^, ^24^, ^29^, ^33^, ^34^, ^35^ rather than solely more favorable tumor biology. After first local treatment, older patients less often underwent an additional local treatment or any subsequent systemic treatment. Whether this reflects appropriate selection of patients with limited disease or undertreatment based on age alone cannot be determined, as our registry does not capture the underlying rationale for treatment decisions.

Two aspects of patient selection add to this uncertainty. Firstly, systematic geriatric screening was lacking, so we cannot verify whether older patients who received less treatment were truly less fit. Secondly, as our cohort was obtained from a tertiary referral registry of patients selected for local treatment, outcomes may overestimate those of the broader population of older patients with CRLM. Prospective studies incorporating geriatric assessment, both within and outside expert centers, are needed to resolve both issues.

Across all age groups, induction systemic treatment was associated with shorter PFS but similar OS, in contrast to other studies reporting improved PFS.^11^, ^36^ This most likely reflects selection bias: patients receiving induction treatment had more and larger CRLM, both established risk factors for earlier recurrence.^26^, ^27^, ^28^ Among patients with synchronous CRLM, those treated without induction treatment had substantially fewer and smaller CRLM, indicating that these patients presented with limited disease burden suitable for upfront local treatment. This is consistent with Dutch/European guidelines reserving induction systemic therapy for downstaging liver lesions or for tumors with poor prognostic factors.^12^

Other important factors influencing treatment allocation and outcomes are RAS/BRAF mutations and MMR status. BRAF mutations are more common in older patients and are associated with worse prognosis.^37^ Molecular data were most often missing in older patients and in patients treated with local treatment only, partly because Dutch guidelines indicate testing only when systemic treatment is considered. However, among patients who received systemic therapy, missing data may reflect inconsistent testing despite guideline recommendations.^9^, ^38^ This is an important limitation that may have caused residual confounding and precludes firm conclusions about the prognostic or predictive value of these markers in our cohort.

Several further limitations apply. Firstly, the retrospective, nonmatched design entails a risk of selection bias and residual confounding despite multivariable adjustment. The study period (2018-2023) spans considerable technical advances in local treatment. However, a prior AmCORE analysis showed stable OS over these years despite improved ablation outcomes,^39^ and PFS did not differ between patients treated in 2018-2021 versus 2022-2023 in our own cohort (*P* = 0.4; data not shown). Secondly, the eoCRC group was small (*n* = 31), limiting statistical power, although this reflects the real-world incidence of CRC in younger patients. Findings should therefore be interpreted with appropriate caution. Thirdly, single-center data, albeit from a large liver referral center, may limit the generalizability. Fourthly, only patients receiving local treatment were included, in line with our study aim, so our findings do not represent the full-CRLM population. Finally, we used multivariable Cox regression as our primary analytical approach, because propensity-score weighting risked unstable estimates, particularly in the small eoCRC subgroup. However, a propensity-score–weighted sensitivity analysis in the aoCRC and loCRC groups yielded consistent results. We did not model the effect of subsequent treatment on survival: a time-fixed covariate risks immortal time bias and a time-dependent approach was not feasible given our sample size and the lack of induction treatment duration data. Larger multicenter studies applying such methods are warranted to validate our results and investigate the effect of subsequent treatments.

Despite these limitations, our detailed, systematically collected data provide valuable insight into an area in which evidence on young patients with CRLM remains relatively scarce. Our findings may serve as a foundation for future multicenter studies in larger and more diverse populations. These future studies may also benefit from emerging approaches such as artificial intelligence-based total tumor volume assessment, which could improve risk stratification and help disentangle the contributions of disease burden and tumor biology to age-related differences in outcomes.

### Conclusion

In patients with CRLM, young individuals more frequently presented with synchronous CRLM and a greater number of metastases at diagnosis, and experienced shorter PFS despite more intensive treatment. Older patients more often presented with metachronous CRLM and a smaller number of liver metastases. OS was similar across age groups despite differences in treatment intensity, suggesting that disease presentation and patient selection, rather than age *per se*, are the principal determinants of outcome. Treatment decisions should be guided by clinical, biological, and disease-specific factors rather than age alone.

## Informed consent

Written informed consent to take part in the study and to publish the article has been obtained from all participants or their legal representatives. The privacy rights of participants have been observed.

## Ethical approval

This study was performed in compliance with relevant laws, regulatory frameworks and guidelines where the research took place.

This study was approved by the METC Amsterdam UMC. (Approval No. METc 2021.01.0121).
