# Supplementary material for: Differences in treatment and outcomes between young and older patients with colorectal cancer liver metastases: an AmCORE-based study
Source: ESMO Gastrointest Oncol. 2026 Nov 9;14:100435. doi: 10.1016/j.esmogo.2026.100435 (PMC13760982; doi:10.1016/j.esmogo.2026.100435)
Supplement: Supplementary file 1 [file mmc1.docx]

# Supplementary

**Table S1. (Neo)adjuvant treatment of primary colon tumors**

|  |  | **Total**  **n=235**  **(100%)** | **<50 years**  **n=19**  **(8.1%)** | **50-69 years**  **n=111**  **(47.2%)** | **≥70 years**  **n=105**  **(44.7%)** | **p-value** |
| --- | --- | --- | --- | --- | --- | --- |
| **(Neo)adjuvant treatment of primary colon tumor** | Neoadjuvant chemotherapy | 13 (5.5%) | 4 (21.1%) | 7 (6.3%) | 2 (1.9%) |  |
|  | Adjuvant chemotherapy | 37 (15.7%) | 0 | 21 (18.9%) | 16 (15.2%) |  |
|  | Chemoradiotherapy | 2 (0.9%) | 0 | 2 (1.8%) | 0 |  |
|  | Radiotherapy | 2 (0.9%) | 1 (5.3%) | 1 (0.9%) | 0 |  |
|  | None | 181 (77%) | 14 (73.7%) | 80 (72.1%) | 87 (82.9%) | 0.003^a^ |

a= Fisher’s exact test; The numbers presented in this table represent the frequency of each treatment modality rather than the absolute number of patients. As some patients received more than one treatment, the sum of the individual treatment counts may exceed the total number of patients in the study.

**Table S2. (Neo)adjuvant treatment of primary rectum tumors**

|  |  | **Total**  **n=115**  **(100%)** | **<50 years**  **n=12**  **(10.4%)** | **50-69 years**  **n=53**  **(46.1%)** | **≥70 years**  **n=50**  **(43.5%)** | **p-value** |
| --- | --- | --- | --- | --- | --- | --- |
| **(Neo)adjuvant treatment of primary rectum tumor** | Neoadjuvant chemotherapy | 8 (7%) | 0 | 5 (9.4%) | 3 (6.1%) |  |
|  | Adjuvant chemotherapy | 2 (1.8%) | 0 | 0 | 2 (4.1%) |  |
|  | Chemoradiotherapy | 38 (33.3%) | 8 (66.7%) | 20 (37.7%) | 10 (20.4%) |  |
|  | Radiotherapy | 28 (24.6%) | 2 (16.7%) | 8 (15.1%) | 18 (36.7%) |  |
|  | None | 38 (33.3%) | 2 (16.7%) | 20 (37.7%) | 16 (32.7%) | 0.029^a^ |

a= Fisher’s exact test; The numbers presented in this table represent the frequency of each treatment modality rather than the absolute number of patients. As some patients received more than one treatment, the sum of the individual treatment counts may exceed the total number of patients in the study.

**Table S3. Post-hoc analyses**

|  | **Initial p-value** | **<50 years compared to 50-69 years** | **<50 years compared to ≥70 years** | **50-69 years compared to ≥70 years** |
| --- | --- | --- | --- | --- |
| **Sex** | 0.040^a^ | 0.867 | 0.247 | 0.084 |
| **ASA** | 0.018^b^ | 0.600 | 0.089 | 0.047 |
| **Comorbidities** | <0.001^b^ | 0.395 | <0.001 | <0.001 |
| **Primary tumor location** | 0.030^a^ | 0.759 | 0.642 | 0.024 |
| **pN stage** | 0.032^a^ | 0.101 | 0.017 | 0.286 |
| **Timing of CRLM diagnosis**  (synchronous vs. (early- and late-) metachronous) | 0.027^a^ | 0.034 | 0.004 | 0.378 |
| **Number of CRLM at diagnosis** | 0.030^c^ | 0.124 | 0.026 | 0.183 |
|  |  |  |  |  |
| **Systemic treatment ever** | <0.001^a^ | 0.137 | <0.001 | <0.001 |
| **Induction chemotherapy prior first local treatment** | <0.001^a^ | 0.052 | <0.001 | 0.001 |

p-values from post-hoc pairwise comparisons between groups. All p-values are adjusted for multiple testing. p<0.05 is considered statistically significant. Abbreviations: ASA = American Society of Anesthesiologists score; pN stage = pathological N stage; CRLM = colorectal liver metastases; a= Pearson’s chi-squared test; b= Fisher’s exact test; c= Kruskal-Wallis test

**Table S4. Induction chemotherapeutic regimen and response**

|  |  | **Total**  **n=119**  **(100%)** | **<50 years n=19**  **(15.1%)** | **50-69 years n=66**  **(55.5%)** | **≥70 years n=34**  **(28.6%)** | **p-value** |
| --- | --- | --- | --- | --- | --- | --- |
| **Chemotherapeutic** | Monotherapy ± bevacizumab^a^ | 4 (3.4%) | 0 | 1 (1.5%) | 4 (11.8%) |  |
| **regimen** | Doublet ± bevacizumab^b^ | 76 (63.9%) | 9 (47.4%) | 41 (62.1%) | 25 (73.5%) |  |
|  | Panitumumab ± doublet^c^ | 14 (11.7%) | 6 (31.6%) | 7 (10.6%) | 1 (2.9%) |  |
|  | Triplet therapy± bevacizumab^d^  Encorafenib-cetuximab | 24 (20.2%)  1 (0.8%) | 4 (21.1%)  0 | 17 (25.8%)  0 | 3 (8.8%)  1 (2.9%) | 0.004^e^ |
| **Best-observed** | Progressive disease | 6 (5%) | 0 | 2 (3%) | 4 (11.8%) |  |
| **response** | Stable disease | 11 (9.2%) | 1 (5.3%) | 7 (10.6%) | 3 (8.8%) |  |
|  | Partial response | 102 (85.7%) | 18 (94.7%) | 57 (86.4%) | 27 (79.4%) |  |
|  | Complete response | 0 | 0 | 0 | 0 | 0.359^e^ |

Categorical variables are reported as absolute number of patients (%); a= capecitabine- 5FU/LV, teysuno ± bevacizumab; b= CAPOX, CAPOX FOLFOX, CAPIRI, CAPIRI-, FOLFIRI, FOLFIRI ± bevacizumab; c= FOLFOX-panitumumab, FOLFIRI-panitumumab, panitumumab; d= FOLFOXIRI ± bevacizumab; e= Fisher’s exact test

**Table S5. Characteristics of patients <50 years old**

|  | **Total n = 31** | **Induction systemic treatment**  **n = 19** | **No induction systemic treatment**  **n = 12** | **p-value** |
| --- | --- | --- | --- | --- |
| **Primary tumor location** | Rectum | 5 (26.3%) | 7 (58.3%) |  |
|  | Left-sided colon | 3 (15.8%) | 3 (25%) |  |
|  | Right-sided colon | 11 (57.9%) | 2 (16.7%) | 0.075^a^ |
| **Number of CRLM at diagnosis** | Median (IQR) | 5 (7) | 2 (2) | <0.001^b^ |
| **Diameter largest CRLM (in mm)** | Median (IQR) | 24 (12.2) | 31.5 (7) | 0.497^b^ |
| **Timing of CRLM diagnosis** | Synchronous | 15 (78.9%) | 7 (58.3%) |  |
|  | Early-metachronous | 3 (15.8%) | 5 (41.7%) |  |
|  | Late-metachronous | 1 (5.3%) | 0 | 0.206^a^ |
| **Molecular profile** | RAS  Wildtype  Mutation  Unknown | 11 (61.1%)  7 (38.9%)  0 | 1 (9.1%)  3 (27.3%)  7 (63.6%) | 0.293^a^ |
|  | BRAF  Wildtype  Mutation  Unknown | 17 (94.4%)  1 (5.6%)  0 | 4 (36.4%)  0  7 (63.6%) | 1.00^a^ |
|  | MMR  MMR proficient  MMR deficient  Unknown | 19 (100%)  0  0 | 11 (91.7%)  0  1 (8.3%) | 1.00^a^ |

Abbreviations: CRLM = colorectal liver metastases; IQR = interquartile range; RAS = rat sarcoma viral oncogene homolog; BRAF = v-Raf murine sarcoma viral oncogene homolog B1; MMR = mismatch repair status; a = Fisher’s exact test; b = Mann-Whitney U test

**Table S6. Characteristics of patients 50-69 years old**

|  | **Total n = 164** | **Induction systemic treatment**  **n = 66** | **No induction systemic treatment**  **n = 98** | **p-value** |
| --- | --- | --- | --- | --- |
| **Primary tumor location** | Rectum | 17 (25.8%) | 36 (36.7%) |  |
|  | Left-sided colon | 14 (21.2%) | 17 (17.3%) |  |
|  | Right-sided colon | 35 (53%) | 45 (45.9%) | 0.330^a^ |
| **Number of CRLM at diagnosis** | Median (IQR) | 3 (5) | 1 (1.2) | <0.001^b^ |
| **Diameter largest CRLM (in mm)** | Median (IQR) | 28 (22) | 21 (20.8) | <0.001^b^ |
| **Timing of CRLM diagnosis** | Synchronous | 47 (71.2%) | 44 (44.9%) |  |
|  | Early-metachronous | 13 (19.7%) | 20 (20.4%) |  |
|  | Late-metachronous | 6 (9.1%) | 34 (34.7%) | <0.001^b^ |
| **Molecular profile** | RAS  Wildtype  Mutation  Unknown | 28 (42.4%)  27 (40.9%)  11 (16.7%) | 21 (23.3%)  25 (27.8%)  44 (48.9%) | 0.690^a^ |
|  | BRAF  Wildtype  Mutation  Unknown | 50 (75.8%)  4 (6.1%)  12 (18.2%) | 44 (49.4%)  2 (2.2%)  43 (48.3%) | 0.684^a^ |
|  | MMR  MMR proficient  MMR deficient  Unknown | 60 (89.6%)  1 (1.5%)  6 (9%) | 80 (85.1%)  2 (2.1%)  12 (12.8%) | 1.00^a^ |

Abbreviations: CRLM = colorectal liver metastases; IQR = interquartile range; RAS = rat sarcoma viral oncogene homolog; BRAF = v-Raf murine sarcoma viral oncogene homolog B1; MMR = mismatch repair status; a = Fisher’s exact test; b = Mann-Whitney U test

**Table S7. Characteristics of patients ≥70 years**

|  | **Total n = 156** | **Induction systemic treatment**  **n = 34** | **No induction systemic treatment**  **n = 122** | **p-value** |
| --- | --- | --- | --- | --- |
| **Primary tumor location** | Rectum | 12 (36.4%) | 38 (31.1%) |  |
|  | Left-sided colon | 5 (15.2%) | 46 (37.7%) |  |
|  | Right-sided colon | 16 (48.5%) | 38 (31.1%) | 0.030^a^ |
| **Number of CRLM at diagnosis** | Median (IQR) | 3 (6) | 1 (1) | <0.001^b^ |
| **Diameter largest CRLM (in mm)** | Median (IQR) | 29 (18) | 22 (15) | 0.037^b^ |
| **Timing of CRLM diagnosis** | Synchronous | 23 (67.6%) | 60 (49.2%) |  |
|  | Early-metachronous | 3 (8.8%) | 22 (18%) |  |
|  | Late-metachronous | 8 (23.5%) | 40 (32.8%) | 0.173^a^ |
| **Molecular profile** | RAS  Wildtype  Mutation  Unknown | 19 (57.6%)  11 (33.3%)  3 (9.1%) | 20 (18%)  18 (16.2%)  73 (65.8%) | 0.462^a^ |
|  | BRAF  Wildtype  Mutation  Unknown | 25 (75.8%)  4 (12.1%)  4 (12.1%) | 38 (34.2%)  1 (0.9%)  72 (64.9%) | 0.155^a^ |
|  | MMR  MMR proficient  MMR deficient  Unknown | 31 (91.2%)  1 (2.9%)  2 (5.9%) | 61 (53.5%)  3 (2.6%)  50 (43.9%) | 1.00^a^ |

Abbreviations: CRLM = colorectal liver metastases; IQR = interquartile range; RAS = rat sarcoma viral oncogene homolog; BRAF = v-Raf murine sarcoma viral oncogene homolog B1; MMR = mismatch repair status; a = Fisher’s exact test; b = Mann-Whitney U test

**Table S8. Characteristics of patients with synchronous and metachronous CRLM**

|  | **Total**  **n = 351** | **Induction systemic treatment**  **n = 119** | **No induction systemic treatment**  **n = 232** | p-value |
| --- | --- | --- | --- | --- |
| **Synchronous CRLM**  Number of CRLM at diagnosis  Diameter largest CRLM (in mm) | **196 patients**  3 (1-6)  25 (18-40) | **85 patients**  7 (4-11)  31 (21-57) | **111 patients**  1 (1-3)  23 (14-33) | <0.001^a^  <0.001^a^ |
| **Metachronous CRLM**  Number of CRLM at diagnosis  Diameter largest CRLM (in mm) | **155 patients**  1 (1-3)  26 (20-34) | **34 patients**  3 (1-8)  32 (24-40) | **121 patients**  1 (1-2)  25 (19-33) | <0.001^a^  0.009^a^ |

a = Mann-Whitney U test; Abbreviations: CRLM = colorectal liver metastases; Variables are reported as median with interquartile range.

## Table S9. Treatment patterns after first local treatment for CRLM, by age group

|  | **Overall  N = 351^a^** | **<50 years  N = 31** | **50-69 years  N = 164** | **≥70 years  N = 156** | **p-value** |
| --- | --- | --- | --- | --- | --- |
| **Treatment after first local treatment**  No subsequent treatment  Directly to palliative systemic therapy  Subsequent local treatment (session 2) | 110 (31%)  88 (25%)  153 (44%) | 4 (13%)  8 (26%)  19 (61%) | 42 (26%)  49 (30%)  73 (45%) | 64 (41%)  31 (20%)  61 (39%) | 0.003^a^ |
| **Total number of CRLM treated in session 2**^c^ | 1 (1-3) | 2 (1-3) | 1 (1-4) | 1 (1-2) | 0.353^b^ |
| **Induction systemic treatment prior to session 2**^c^  Yes  No | 56 (37%)  97 (63%) | 11 (58%)  8 (42%) | 32 (44%)  41 (56%) | 13 (21%)  48 (79%) | <0.001 |
| **Total number of CRLM treated in session ≥3**^d^ | 2 (1-5) | 4 (3-6) | 3 (1-4) | 2 (1-3) | 0.200^b^ |
| **Palliative systemic therapy during follow-up**^e^  Yes  No | 62 (43%)  201 (57%) | 8 (35%)  15 (65%) | 34 (30%)  81 (70%) | 20 (16%)  105 (84%) | 0.016^a^ |
| **Total number of systemic treatment sessions** | 1 (0-2) | 2 (1-3) | 1 (0-2) | 0 (0-1) | <0.001^b^ |
| **Total number of subsequent local treatment sessions** | 0 (0-1) | 1 (0-2) | 0 (0-1) | 0 (0-1) | 0.027^b^ |

Data presented as median (IQR) for continuous variables and n (%) for categorical variables. a = Fisher’s exact test; b = Kruskal-Wallis rank sum test; c = N=153 patients; d = N=69 patients; e = Restricted to patients who did not proceed directly to palliative-intent systemic therapy after their first local treatment (N=263/351). "Yes" indicates that a systemic treatment cycle occurring later in the treatment course was not followed by (further) local treatment, marking transition to palliative-intent therapy.

Abbreviations: CRLM = colorectal liver metastases; IQR = interquartile range

**Figure S1. PFS across all age groups in patients with and without induction chemotherapy**

**a**

**b**

**c**

Kaplan-Meier curves of progression-free survival (PFS), comparing patients who received induction chemotherapy with patients who did not receive induction chemotherapy. Compared with log-rank test. Red indicates patients who did not receive induction chemotherapy. Blue indicates patients who did receive induction chemotherapy. Number of events are per patient; a = PFS of patients <50 years; b = PFS of patients 50-69 years; c = PFS of patients ≥70 years

**Table S10. Median PFS of all age categories.**

| Age categories | Median PFS (95% CI) |
| --- | --- |
| <50 years  Synchronous CRLM (n=22)  Metachronous CRLM (n=9) | 4.4 (3.0-7.6)  4.3 (3.0-7.6)  4.8 (2.9-NA) |
| 50-69 years  Synchronous CRLM (n=91)  Metachronous CRLM (n=73) | 6.6 (5.3-8.0)  6.9 (5.8-8.9)  5.3 (4.8-10.2) |
| ≥70 years  Synchronous CRLM (n=83)  Metachronous CRLM (n=73) | 7.3 (5.9-9.3)  6.6 (5.5-10.2)  7.6 (5.2-12.9) |

Median PFS with corresponding 95% CI stratified by age groups and further subdivided by synchronous and metachronous CRLM. Metachronous CRLM consist of both early and late metachronous CRLM.

**Figure S2. OS of patients with and without induction chemotherapy**

Kaplan-Meier curves of OS, comparing patients who received induction chemotherapy with patients who did not receive induction chemotherapy, p = 0.66. Red indicates patients who did not receive induction chemotherapy. Blue indicates patients who did receive induction chemotherapy.

**Table S11. Associations of variables with survival**

|  | **Univariable analysis** | **p-value** | **Multivariable analysis** | **p-value** |
| --- | --- | --- | --- | --- |
|  | **Unadjusted HR**  **(95% CI)** |  | **Adjusted HR**  **(95% CI)** |  |
| **Age categories** |  | 0.2 |  | 0.596 |
| <50 years  50-69 years | 0.70 (0.33-1.46)  (Reference) |  | 0.78 (0.34-1.82)  (Reference) |  |
| **≥**70 years | 1.25 (0.86-1.81) |  | 1.19 (0.72-1.96) |  |
| **Sex** |  | 0.959 |  | 0.804 |
| Male | (Reference) |  | (Reference) |  |
| Female | 0.99 (0.67-1.45) |  | 0.94 (0.56-1.57) |  |
| **Comorbidities** |  | 0.047 |  | 0.176 |
| None | (Reference) |  | (Reference) |  |
| Minimal | 1.36 (0.90-2.04) |  | 1.24 (0.72-2.12) |  |
| Major | 2.09 (1.17-3.73) |  | 2.03 (0.99-4.20) |  |
| **Primary tumor location** |  | 0.003 |  | 0.441 |
| Rectum | (Reference) |  | (Reference) |  |
| Right-sided | 1.42 (0.90-2.23) |  | 1.16 (0.64-2.09) |  |
| Left-sided | 2.26 (1.40-3.66) |  | 1.50 (0.79-2.86) |  |
| **cT/pT stage**  T1  T2  T3  T4 | (Reference)  0.14 (0.04-0.50)  0.30 (0.12-0.76)  0.48 (0.18-1.26) | 0.006 | (Reference)  0.28 (0.05-1.59)  0.48 (0.10-2.18)  0.74 (0.15-3.67) | 0.219 |
| **cN/pN stage**  N0  N1  N2 | (Reference)  1.43 (0.89-2.30)  2.19 (1.31-3.66) | 0.012 | (Reference)1.46 (0.80-2.68)  2.47 (1.33-4.60) | 0.015 |
| **Molecular profile** |  |  |  |  |
| RAS |  | 0.181 |  | 0.062 |
| Wildtype | (Reference) |  | (Reference) |  |
| Mutation | 1.53 (0.97-2.40) |  | 1.40 (0.77-2.52) |  |
| Unknown | 1.23 (0.77-1.96) |  | 0.21 (0.04-1.24) |  |
| BRAF |  | 0.15 |  | 0.101 |
| Wildtype | (Reference) |  | (Reference) |  |
| Mutation | 2.51 (1.08-5.80) |  | 2.00 (0.45-8.97) |  |
| Unknown | 1.14 (0.77-1.70) |  | 5.50 (0.99-30.51) |  |
| MMR |  | 0.289 |  | 0.270 |
| MMR proficient | (Reference) |  | (Reference) |  |
| MMR deficient | 2.46 (0.90-6.71) |  | 5.68 (0.65-50.05) |  |
| Unknown | 1.10 (0.69-1.76) |  | 1.34 (0.66-2.70) |  |
| **Timing of CRLM diagnosis** |  | 0.192 |  | 0.036 |
| Synchronous CRLM | (Reference) |  | (Reference) |  |
| Early metachronous CRLM | 1.44 (0.91-2.28) |  | 2.02 (1.11-3.66) |  |
| Late metachronous CRLM | 1.36 (0.88-2.10) |  | 1.79 (1.00-3.19) |  |
| **Diameter of largest tumor at diagnosis** | 1.00 (0.99-1.01) | 0.593 | 1.00 (0.99-1.01) | 0.929 |
| **Number CRLM at diagnosis** | 1.01 (0.97-1.06) | 0.506 | 1.02 (0.97-1.08) | 0.424 |

Abbreviations: HR = hazard ratio; 95% CI = 95% confidence interval; CRLM = colorectal liver metastases; RAS = rat sarcoma viral oncogene homolog; BRAF = v-Raf murine sarcoma viral oncogene homolog B1; MMR = mismatch repair status; cT stage clinical T stage; pT stage = pathological T sage; cN stage = clinical N stage; cT stage = clinical T stage

**Table S12. Variables influencing the decision for induction systemic treatment**

|  | **Odd’s ratio (95% CI)** | **p-value** |
| --- | --- | --- |
| **Age categories** |  |  |
| <50 years  50-69 years | 1.78 (0.57-5.63)  (Reference) | 0.321 |
| **≥**70 years | 0.44 (0.18-1.05) | 0.064 |
| **Sex** |  |  |
| Male | (Reference) |  |
| Female | 1.28 (0.58-2.79) | 0.536 |
| **Comorbidities** |  |  |
| None | (Reference) |  |
| Minimal | 1.41 (0.60-3.34) | 0.429 |
| Major | 0.62 (0.13-2.34) | 0.495 |
| **Primary tumor location** |  |  |
| Rectum | (Reference) |  |
| Right-sided | 1.25 (0.54-2.95) | 0.600 |
| Left-sided | 0.63 (0.21-1.75) | 0.372 |
| **Molecular profile** |  |  |
| RAS |  |  |
| Wildtype | (Reference) |  |
| Mutation | 0.92 (0.34-2.46) | 0.863 |
| Unknown | 0.05 (0.00-0.94) | 0.170 |
| BRAF |  |  |
| Wildtype | (Reference) |  |
| Mutation | 3.2 (0.40-36.10) | 0.279 |
| Unknown | 11.08 (0.66-1371.27) | 0.109 |
|  |  |  |
| MMR |  |  |
| MMR proficient | (Reference) |  |
| MMR deficient | 0.27 (0.01-5.17) | 0.420 |
| Unknown | 0.90 (0.30-2.77) | 0.854 |
| **Timing of CLRM diagnosis** |  |  |
| Synchronous CRLM | (Reference) |  |
| Early metachronous CRLM | 0.61 (0.22-1.60) | 0.318 |
| Late metachronous CRLM | 0.54 (0.21-1.33) | 0.181 |
| **Diameter of largest CRLM at diagnosis** | 1.02 (1.00-1.05) | <0.001 |
| **Number CRLM at diagnosis** | 1.56 (1.36-1.82) | <0.001 |

Abbreviations: OR = odd’s ratio; 95% CI = 95% confidence interval; CRLM = colorectal liver metastases; RAS = rat sarcoma viral oncogene homolog; BRAF = v-Raf murine sarcoma viral oncogene homolog B1; MMR = mismatch repair status
